# Systemic inflammation mediating the relationship between lifestyle factors and musculoskeletal pain: a systematic review

**DOI:** 10.3389/fpain.2026.1755744

**Published:** 2026-04-01

**Authors:** David Josefsson, Louise Danielsson, Max Jakobsson

**Affiliations:** Department of Health and Rehabilitation, Institute of Neuroscience and Physiology, Sahlgrenska Academy, University of Gothenburg, Gothenburg, Sweden

**Keywords:** lifestyle factors, mediation analysis, musculoskeletal pain, psychological factors, systemic inflammation

## Abstract

**Background:**

A range of studies have shown that psychological and lifestyle factors are associated with musculoskeletal pain. The factors also correlate with persistent systemic inflammation. Since systemic inflammation is also present in numerous conditions involving musculoskeletal pain, it is of interest to examine whether systemic inflammation mediates the relationship between psychological and lifestyle factors and musculoskeletal pain.

**Methods:**

A systematic review was conducted according to the PRISMA guidelines. The PubMed, Scopus and PsycINFO databases were searched for studies examining systemic inflammation as a mediating factor between psychological and/or lifestyle factors and musculoskeletal pain. Studies were grouped into three themes of influencing factors; sleep disturbances, obesity and psychological factors. A risk of bias assessment tool for mediation studies was used to assess methodological quality. A narrative synthesis of outcomes was performed.

**Results:**

Twenty-one articles were included in the review. Evidence derived primarily from moderate-quality studies suggested small but significant mediating effects of systemic inflammation on the relationship between sleep disturbance and musculoskeletal pain, as well as between obesity and osteoarthritis. In contrast, no consistent mediating effects were observed for psychological factors. However, findings from studies of low to moderate quality indicated potential interaction effects, suggesting that psychological factors may lead to pain in the presence of systemic inflammation.

**Conclusion:**

Systemic inflammation may represent a biological pathway linking sleep disturbances and obesity to musculoskeletal pain, likely operating alongside other mediating factors. For psychological factors, systemic inflammation appears to act as an interacting variable rather than a direct mediator of pain. Given the reliance on cross-sectional data in current research, longitudinal studies are essential to confirm these causal mechanisms and to evaluate the clinical significance of targeting systemic inflammation in pain management.

## Background

Musculoskeletal pain represents a significant burden on society and widespread suffering for the individual. Musculoskeletal conditions are the leading cause of disability worldwide, and the number of affected individuals is rapidly increasing (1).

The importance of psychological and lifestyle factors in the development of musculoskeletal pain is well established (2–5). These are states, conditions, and behaviours linked to general health, such as overweight/obesity, smoking, physical inactivity, sleep disturbances, socioeconomic status, psychological stress, anxiety and depression (2–8). They may also encompass more subjective psychological factors such as traumatic experiences, adverse childhood experiences (ACEs), and lack of social support (9).

It seems that the same psychological and lifestyle factors that are linked to general health are also linked to musculoskeletal pain. Considering this, the increasing prevalence of musculoskeletal disorders might reflect generally declining health in Western countries (10, 11).

There is a complex interaction between lifestyle and pain and it is unclear whether a unifying link across conditions exists (12). “Systemic inflammation” or “chronic low-grade inflammation” has been linked to both psychological and lifestyle factors, as well as musculoskeletal pain (13–15).

In contrast to classical or “overt” inflammation as a response to tissue injury or infection, the phenomenon of systemic inflammation is characterised by a significantly more moderate and less focused response (16, 17). It is primarily measured as increased serum levels of acute-phase proteins, such as C-reactive protein (CRP) and proinflammatory cytokines such as some of the interleukins (IL-1β, IL-6) and tumour necrosis factor alpha (TNF*α*). This state of systemic inflammation resides somewhere in between the classical signs of pathology and those of optimal health. Inflammatory markers are significantly elevated, while not being on par with the expected levels during infectious disease or major traumatic injury (16, 18). Although being increasingly mentioned in the literature, there is still a lack of consensus regarding the proper definition, or meaning of systemic inflammation (13, 16, 18). It has, however, been linked to a plethora of conditions associated with poor health and with chronic pain (15, 18). The same psychological and lifestyle factors that correlate with musculoskeletal pain have also been reported to correlate with systemic inflammation (13, 14, 19–22).

Questions remain about cause-and-effect relationships in this field. It is possible that the vast number of psychological and lifestyle factors associated with musculoskeletal pain might have a causative role through the induction of an inflammatory state. The observed bidirectional correlations suggest this; not only do systemic inflammation and musculoskeletal pain correlate with the same psychological and lifestyle factors (3–5, 7, 8, 13) but systemic inflammation also correlates with musculoskeletal pain conditions (15). Elevated CRP and/or increased serum levels of proinflammatory cytokines have been observed in various somatic pain conditions, not normally considered “inflammatory” (15). Examples are back and neck pain (23, 24), headaches (25), fibromyalgia (26), temporomandibular joint disorders (TMD) (27), peripheral neuropathies (28), chronic regional pain syndrome (CRPS) (29), and others (15). It is a reasonable hypothesis that these are not mere correlations, but that certain lifestyle and psychological factors stimulate inflammatory activity, which in turn influences nociceptive pathways.

Moreover, evidence supporting the physiological basis of this hypothesis is steadily accumulating. Circulating proinflammatory cytokines have the potential to create pain in several distinct ways. They may act directly on nociceptive neurons through their respective receptors or indirectly through the induction of prostaglandin synthesis (30, 31). They mediate muscle and joint hyperalgesia by sensitising nociceptors in peripheral nerve terminals (32–34). Additionally, they have been found to contribute to central sensitisation through direct receptor-mediated effects on afferent nociceptive pathways in spinal dorsal horn and dorsal root ganglia, but also act indirectly through glial activation in the central nervous system (CNS) (34–37). Experimentally induced systemic inflammation via the injection of lipopolysaccharides (LPS), a well-established model for studying inflammatory properties, decreases pain thresholds in somatic structures across multiple modalities in humans (38–40). Recent hypotheses propose effects of systemic inflammation on both pain and local tissue quality and a reciprocal relationship between local and systemic inflammation (41).

Present clinical guidelines for chronic pain involve a biopsychosocial approach (12). Thus, focus has shifted from treating local pain sites to emphasizing the whole person in their lived context. In line with this, further investigations of systemic inflammation and lifestyle in relation to pain might provide important details to better understand the complexity of pain. Moreover, it is important to identify factors that cause the transition of acute to chronic pain (12). Elucidating how risk factors for chronification (such as lifestyle) influence pain perception, potentially via neuroimmune pathways, would advance knowledge in the field. If a unifying mechanistic link tying lifestyle and general health to musculoskeletal pain can be demonstrated in research, it is likely to influence clinical practice as well. It could to a greater extent motivate intervention strategies and public health investments targeting lifestyle factors. It could also provide a helpful narrative for better patient communication.

There are few direct studies on the relationship between influencing factors, pain conditions, and systemic inflammation. Attempts have been made to study individual links between these three variables, such as whether systemic inflammation mediates the effect of sleep disturbances on temporomandibular joint pain (42). To the author's knowledge there is currently no systematic summary of studies analysing inflammatory activity as the mediating factor between influencing factors and pain conditions.

## Aim

The aim of this study was to evaluate the support for systemic inflammation as a mediating factor between psychological and lifestyle factors and musculoskeletal pain.

## Methods

The study was conducted as a systematic literature review according to the PRISMA guidelines (43), with a narrative synthesis applied due to the expected heterogeneity of the study designs and outcomes.

### Search strategy

Published articles on the topic of systemic inflammation as a mediating factor between psychological and/or lifestyle factors and somatic pain were identified by database searches.

#### Inclusion criteria

- Studies concurrently examining all three of the variables musculoskeletal pain, systemic inflammation, and influential (psychological or lifestyle) factors
- Studies with the relevant terms in the title, abstract or keywords
- Studies explicitly testing mediation or providing information that allowed assessment of mediating effects
- Studies measuring serum or urinary levels of inflammatory markers
- Studies measuring either pain directly or through the occurrence of painful conditions

#### Exclusion criteria

- Animal studies
- Studies on children
- Studies involving autoimmune diseases or non-musculoskeletal pathologies
- Studies involving traumatic injuries
- Studies including surgical interventions
- Secondary research (reviews, meta-analyses and editorials)

#### Information sources

The databases PubMed, Scopus and PsycINFO were searched for relevant articles. Searches were made in PubMed (October 29), Scopus (November 21) and PsycINFO (December 2) 2024.

Searches included four search blocks with multiple terms for each of the categories “influential factors” (psychological and lifestyle), “systemic inflammation” and “somatic pain” and one for terms concerning mediation. A fifth block excluded irrelevant medical areas/conditions and other review articles. For a full review of the search strategy, see Supplementary Files S1.

The searches were limited to peer-reviewed articles published between 2004 and 2024, written in English, involving human subjects.

### Study selection

Based on title and abstract, all eligible studies were extracted from each database. After duplicates were removed, the remaining studies were screened for inclusion. During the screening process, three groups of influential factors were in a clear majority, while other factors were only sporadically studied. Therefore, we decided to exclude studies investigating other factors than the three main themes (sleep, obesity and psychological factors) for increased homogeneity in the analysis. In total, 6 studies were excluded in this way (44–49), before further considering their eligibility in relation to study design etc. These included single studies examining each and every one of the factors; socioeconomic status, aging, type 2 diabetes, drug abuse, static posture exposure and demographic factors including education level and smoking.

For the last phase of the screening process, articles were read in full-text. Most of the studies excluded in this phase specifically lacked data on mediation effects. Screening and study selection were performed by the first author (DJ). Any uncertainties regarding study eligibility were resolved through consensus discussions with the other two authors (LD, MJ).

### Data extraction

Data extraction was performed by a single reviewer (DJ). The extracted data included study characteristics (e.g., author, year, country, study design), participant demographics, types of psychological and lifestyle factors, specific systemic inflammatory biomarkers, and pain outcomes. To ensure accuracy and consistency, any ambiguities or uncertainties encountered during the extraction process were resolved through discussion and consensus with the other authors.

### Study risk of bias assessment

The analysis was divided into two parts. First, a review of the methodological quality of each included study; and second, a synthesis of their respective outcomes. For this work, a tool for reviewing mediation studies developed by Mansell et al. (50) was used in a specific version adapted for observational studies by Lee et al. (51). This tool was selected to evaluate important conditions for mediation. The assessment includes seven items: [1] use of a theoretical framework; [2] psychometric properties of mediator and outcome variables; [3] statistical power to detect indirect effects; [4] use of appropriate statistical methods for mediation (e.g., SEM or bootstrapping); [5] whether changes in the mediator preceded the outcome; [6] whether changes in the predictor preceded the mediator; and [7] adjustment for potential confounders. The tool can be accessed in Supplementary Files S2.

Each item was scored binarily (yes/no), resulting in a total quality score of 0–7. Studies were categorized as low (0–3), moderate (4–5), or high quality (6–7). Following the recommendations of Fritz and MacKinnon (45), studies with sample sizes sufficiently large to acquire adequate power for mediation were also credited for item 3. To provide a nuanced evaluation beyond the numerical score, qualitative commentaries were added regarding specific strengths or weaknesses, such as the breadth of confounder adjustment or sample characteristics.

To test the assessment tool for heterogeneity in application, all the authors independently read and assessed three of the articles. The results were then compared and discussed in relation to possible ways of interpreting the items of the assessment tool. After this calibration stage, the first author performed the quality assessments of the remaining articles (52).

### Data synthesis

The included studies were divided into three groups reflecting the influential factors studied, being sleep, obesity and psychological factors as described above (see Tables 1–3).

**Table 1:** Studies on the relationship between sleep, musculuskeletal pain and systemic inflammation (*N* = 6).

Study	Country	Population	Influencing factor	Pain variable	Infl. markers	Outcome
Haack et al. (53)	Israel	24 healthy subjects	*Sleep deprivation*	*Pain intensity*	PGE2	Sleep deprivation correlates temporally with elevated PGE2-production and proportionally with pain
Intervention		Age 21–55				
		29% females	88 h wakefulness vs. 8 h sleep/night	VAS ratings of spontaneous pain including: headache, back-, muscle-, joint-, stomach- and generalized body pain		
Haack et al. (54)	Israel	18 healthy subjects	* Sleep deprivation*	*Pain intensity*	IL-6, CRP, sTNF-R p55, PGE2	Sleep loss correlates temporally with increased IL-6 levels and and proportionally with pain
Intervention		Age 21–40	4 h vs. 8 h sleep/night			
		33% females		Ratings of “bodily discomfort” including: headache, back-, muscle-, joint-, stomach- and generalized body pain		
Hodges et al. (55)	UK	17,642 cMSK-pain	“*Sleep score”*	*MSK-pain*	CRP	CRP may partially mediate the association between MSK-pain and sleep score
		11,962 acute MSK-pain	Duration, insomnia, chronotype, snoring, daytime sleepiness	Acute MSK-pain vs. Chronic MSK-pain vs. Painfree controls		
Cross-sectional		29,604 controls				
		Mean age 55.7				
		47% females				
Irwin et al. (56)	US	95 healthy adults	* Sleep-disruption*	*Heat –pain tresholds (hPTH)*	IL-6, TNF*α*	Sleep disturbance is associated with decreased hPTH mediated by elevated IL-6 and TNF*α* levels
Cross-over RCT		Mean age 27.8	Forced awakenings + Measures with polysomnography			
		54% females				
Matre et al. (57)	Norway	23,223 Workers	* Sleep disturbance*	Chronic MSK-pain present/absent	CRP	Shift-work is associated with cMSK pain and number of pain sites, partly mediated by elevated CRP-levels
Cross-sectional		Age 18–70	Shift-work yes/no			
		56% females				
Saravaanan et al. (58) Cross-sectional	US	67 CLBP patients	* Sleep disturbance*	*Pain severity in Chronic low back pain (cLBP)*	IL-6	Sleep disturbance is associated with lack of social support, IL-6 and pain. Sleep disturbance correlates with proportional increases in pain and IL-6 levels. Sleep disturbance mediates the effect of social support on pain.
			Pittsburgh Sleep Quality Index (PSQI)			
		Median age 59	*Social support*	Brief pain inventory (BPI)		
		73% females	Social provisionist scale (SPS)			

**Table 2:** Studies on the relationship between obesity, musculoskeletal pain and systemic inflammation (*N* = 9).

Study	Country	Population	Influencing factor	Pain variable	Infl. markers	Outcome
Dai et al. (59)	US	Sample 1: 2,876 (861 knees)	BMI	*Symptomatic knee OA (SKOS)* Knee pain last 30 days yes/no combined with radiological OA	hsCRP	Less dietary fiber is associated with higher incidence of knee OA mediated through CRP via increased BMI
		Mean age 61				
Longitudinal		55% females				
		Sample 2: 971 (143 knees)	Dietary fiber intake			
		Mean age 54				
		54% females				
Eslami et al. (60)	US	667 elderly	BMI	*Pain intensity and pain interference*	hsCRP	Obesity was associated with increased pain, partly mediated by increased hsCRP-levels in women but not in men(!)
Cross-sectional		Mean age 79		Bodily pain subscale from 36 item short form health survey (SF-36)		
		61% females				
Fowler-Brown et al. (61)	US	653 elderly	BMI	*Knee OA*	Leptin	∼half of the increased risk of OA from elevated BMI was mediated by increased leptin levels
Cross-sectional		Mean age 78		Presence/Absence		
		63% females		Physcial examination using the American College of Rheumatology (ACR) clinical criteria		
Gløersen et al. (62)	Norway	281 Hand OA patients	BMI	*Pain intensity in hand-OA patients* Pain ratings (NRS) for hand-/foot-/knee- and hip pain. *P*ainful total body joint count, pain pressure thresholds (PPT)	hs-CRP, Leptin	Increased BMI were associated with more pain (all kinds) and partially mediated by hs-CRP (painful total body joint count) and leptin (hands only)
Cross-sectional		Age 40–70 Median 61				
		89% females				
Huebner et al. (63)	US	169 obese OA patients	Weight/ Weightloss	*Pain and disaility (OA patients)*	hsCRP, IL-1, IL-6, IL-8, TNFα	Weight loss resulted in ↓IL-6 and leptin levels, which in turn was associated with ↓WOMAC pain
Intervention		Mean age 59		*W*estern Ontario and McMaster Universities Arthritis Index (WOMAC)		
		89% females				
Luo et al. (64)	China	6,497 subjects	BMI	*Knee pain*	CRP	BMI was associated with elevated CRP and knee pain. Obesity mediates the association between BMI and CRP
Cross-sectional		Mean age 44		Miscellaneous Pain Questionnaire (MPQ)		
		48% females				
Perera et al. (65)	UK	Sample 1: 826	Weight, BMI, Total fat mass (TFM)	*Back pain*	CRP, IL-6, TNFα	Increased weight, BMI and TFM was directly associated with increased risk of back pain, not mediated by any measures of inflammatory markers
		Mean age: 60.6				
Cross-sectional and prospective		Sample 2: 645		Presence/Absence of episode in the last year		
		Mean age: 67.2				
		100% females				
Ray et al. (66)	US	407 elderly	BMI	*Pain frequency and intensity*	hsCRP	Central obesity is independently associated with pain, not explained by hsCRP or insulin resistance
Cross-sectional		Mean age 80		Total Pain Index (TPI)		
		62% females				
Yan et al. (67)	China	23,308 subjects	BMI, Abdominal obesity (Waist circumference)	*OA*	HDLP, CRP	Obesity was associated with increased risk of OA partly mediated by HDLP and CRP
Cross-sectional		2,180 OA-patients		Prescence of OA diagnosed by a professional		
		60% >60 years old)				
		63% females				

**Table 3:** Studies on the relationship between psychological factors, musculoskeletal pain and systemic inflammation (*N* = 6).

Study	Country	Population	Influencing factor	Pain variable	Infl. markers	Outcome
Andres-Rodriguez et al. (68)	Spain	70 fibromyalgia patients	Mindfullness-based stress reduction (MBSR)	*Clinical severity of fibromyalgia*	Ratio of IL-6, CXCL8, hs-CRP and IL-10	MBSR reduced severity of FM while simultaneously changing ratio of pro- and antiinflammatory markers (CXCL8/IL-10). Reduced FM-symptoms was associated with sustained IL-10 levels compared to controls
Intervention (RCT)		Mean age 53		Revised fibromyalgia impact questionnaire (FIQR)		
		100% females				
Banafa et al. (69)	Finland	8,028 adults	Depressive symptoms [Beck Depression Inventory (BDI)]	*Temporomandibular pain*	hs-CRP	More depression (BDI-scores) and elevated CRP correlated with more pain. No mediation effect by hsCRP
Cross-sectional and longitudinal		All ages		Presence/Absence TMJ and masticatory muscles (MM) pain on palpation		
		55% females				
Belitardo de Oliveira et al. (70)	Brazil	7,644 adults	Psychosocial job stress [Swedish demand control-support questionnaire (DCSQ)]	*Migraine and Tension-type headache (TTH)*	Hs-CRP, GlycA	Jobb-stress was associated with migraine, not mediated by hsCRP or GlycA. Jobb-stress was associated with TTH and a protective effect was seen from physical activity partly mediated by decreased GlycA levels
Cross-sectional		Mean age 49				
		54% females		Headache questionnaire		
Dalachek et al. (71)	UK	24,164 adults	Anxiety and adverse childhood events (ACE) (Online questionnaire)	*Chronic pain*	CRP	Frequency of childhood abuse significantly interacted with CRP to predict pain. CRP predicts pain during anxiety but not without.
Cross-sectional		Age 40–69				
		61% females		Self-reported pain >3 months rated for 8 body parts		
Graham et al. (72)	US	113 caregivers	Hostility (Cook Medley Hostility scale), Chronic stress (Caregiver of a relative)	*General body pain (Presence/intensity)*	CRP, IL-6	Pain and CRP correlates for caregivers but not controls, Hostility and CRP were associated but equal between groups.
		101 non-caregivers				
Longitudinal		Mean age 69		Bodily pain subscale from SF-36		
		71.5% females				
Poleshuck et al. (73)	US	106 patients	Depressive symptoms [Center for Epidemiological Studies Depression Scale-Revised (CES-D-R)]	*Pain intensity and interference*	IL-6	Increased IL6 levels was associated with more pain in depressed patients but not healthy controls
Cross-sectional		Mean age 52		Bodily pain subscale from SF-36		
		78% females				

Because of the heterogeneity in study designs and outcome measures, a meta-analysis was not considered feasible. Therefore, a narrative synthesis of the studies was conducted, including the relative weight of different study designs, population sizes and the results from the quality review. Similarities and discrepancies across studies and their respective outcomes were compared, evaluated and described. This process included additional groupings where possible, so as to compare similar conditions (e.g., osteoarthritis vs. back pain) and markers of inflammation (e.g., CRP vs. IL-6) and the complementary value of cross-sectional vs. longitudinal study designs for examining the same variables.

Where mediation effects were found, the magnitude (i.e., percentage of correlation due to the mediating variable) was considered along with statistical significance.

## Results

The initial search strategy provided a total of 1,920 studies. The searches in PubMed, Scopus and PsycINFO provided 1,102, 1,559, and 81 studies, respectively. After 822 duplicates had been removed, 1,098 unique studies were screened for potential inclusion in the review.

**Figure 1:**
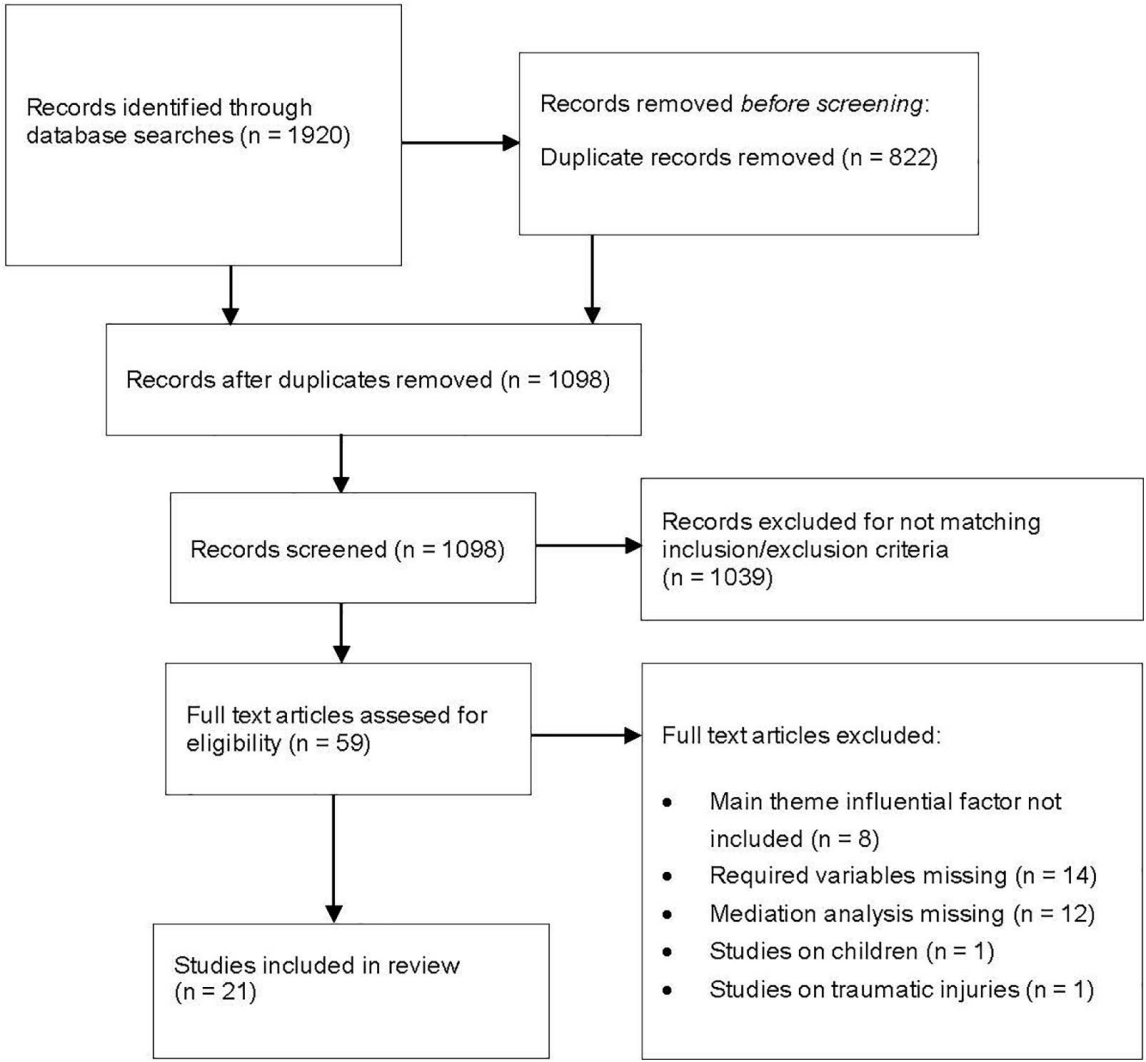
Study flowchart (adapted from PRISMA).

Following the stepwise screening and selection process, 1,077 studies were excluded, see Figure 1 for details. After the full screening and selection process, a total of 21 articles were included in the review (for quality assessment and synthesis of results) (53–73).

Of the included studies, six examined sleep (53–58), nine examined obesity (59–67) and six examined psychological factors (68–73) (Tables 1–3).

Of the 21 studies, 12 had a cross-sectional design, seven were longitudinal/intervention studies and two used a combination of cross-sectional and longitudinal data. Most study samples consisted of middle-aged or elderly participants, and there were more women than men (∼64% females). Musculoskeletal pain was measured in various ways, including self-reported ratings of intensity, pain thresholds and/or the presence of specific conditions such as osteoarthritis (OA) or back pain. The most measured inflammatory markers were CRP/hsCRP (*n* = 16) and IL-6 (*n* = 8).

### Quality assessment

In the quality assessment two of the 21 studies had low quality, 15 had moderate quality, and four had high quality (Tables 4–6). Seven studies had longitudinal designs, whereas the other 14 were cross-sectional and, as such, could not establish temporality between exposure, mediator and outcome. Most studies received lower quality scores because their cross-sectional design did not satisfy the temporality criteria included in two items of the quality assessment tool. The majority of studies failed to report a power calculation, but most of them (*n* = 14) had large enough sample sizes to ensure adequate power (55–57, 59, 60, 62–67, 69–71). For a detailed report of the quality scores, see Supplementary Files S3.

**Table 4:** Quality assessments. Sleep (*N* = 6).

Atuhor	Study design	Quality score	Quality group	Commentary
Haack et al. (53)	Intervention	4	Moderate quality	Highly controlled
Haack et al. (54)	Intervention	4	Moderate quality	Highly controlled
Hodges et al. (55)	Cross-sectional	5	Moderate quality	Large sample
Irwin et al. (56)	Intervention	6	High quality	
Matre et al. (57)	Cross-sectional	4	Moderate quality	Large sample
Saravaanan et al. (58)	Cross-sectional	5	Moderate quality	

**Table 5:** Quality assessments. Obesity (*N* = 9).

Author	Study design	Quality score	Quality group	Commentary
Dai et al. (59)	Longitudinal	7	High quality	Well controlled
Eslami et al. (60)	Cross-sectional	5	Moderate quality	
Fowler-Brown et al. (61)	Cross-sectional	4	Moderate quality	Partially low power
Gløersen et al. (62)	Cross-sectonal	5	Moderate quality	
Huebner et al. (63)	Intervention	6	High quality	
Luo et al. (64)	Cross-sectional	5	Moderate quality	Large sample
Perera et al. (65)	Cross-sectional + Longitudinal	6	High quality	
Ray et al. (66)	Cross-sectional	4	Moderate quality	
Yan et al. (67)	Cross-sectional	5	Moderate quality	Large sample

**Table 6:** Quality assessment. Psychological factors (*N* = 6).

Author	Study design	Quality score	Quality group	Commentary
Andres-Rodriguez et al. (68)	Intervention	3	Low Quality	
Banafa et al. (69)	Cross-sectional + Longitudinal	5	Moderate quality	Large sample
Belitardo de Oliveira et al. (70)	Cross-sectional	5	Moderate quality	Large sample
Dalecheck et al. (71)	Cross-sectional	4	Moderate quality	Large sample
Graham et al. (72)	Longitudinal	4	Moderate quality	
Poleshuck et al. (73)	Cross-sectional	3	Low Quality	

### Results of syntheses

#### Sleep as the predictor variable

Three studies in this group used adequate statistical methods to detect mediation effects (55–57). Two of those were cross-sectional studies (55, 57) of moderate quality, with large samples. Both of these studies found that reduced sleep quality was associated with more frequent pain, partly mediated by elevated CRP levels, but through different exposures. Hodges et al. (55) used a sleep score comprised of several items, while Matre et al. (57) substituted shift-work for sleep quality. The mediation effects were in both instances small, but significant, and in the study by Matre et al. (57), there was a stronger association at higher levels of CRP, indicating a dose-response relationship. The third study by Irwin et al. (56) was an intervention study of high quality on healthy, pain-free subjects. This study demonstrated reduced pain thresholds in response to sleep disruption, partly (19%) mediated by elevated levels of IL6 and TNF*α*. Although causality is claimed in all three of these studies, neither the study by Hodges et al. (55) nor Matre et al. (57) can establish temporality or direction of correlation, due to their cross-sectional designs.

The remaining three studies showed indirect indications of mediation effects, but without the proper statistical analyses. One was a cross-sectional study by Saravaanan et al. (58) on low back pain patients that demonstrated more severe pain with greater sleep disturbance (by Pittsburgh Sleep Quality Index), possibly mediated by elevated IL-6 levels, due to proportional increases.

The other two were intervention studies involving healthy, previously pain-free subjects, both by Haack et al. (53, 54), with similar designs and strictly controlled variables. Participants were monitored at a research centre, reducing the risk of confounders. The intervention groups were deprived of sleep either completely (53) or by 50% (54) compared to the control group. Both studies demonstrated a significant dose-response relationship, where inflammatory markers and pain intensity co-varied synchronously with accumulated sleep loss. Specifically, in the group experiencing complete sleep deprivation, levels of prostaglandin E2 (PGE2) metabolites and spontaneous pain increased proportionally and gradually with accumulated sleep deprivation. In the group experiencing 50% sleep deprivation, IL-6 levels and pain increased over time and proportionally to each other, indicating a possible mediation effect. All six studies on sleep found a relationship between altered sleep (quality, time, disruption) and musculoskeletal pain, where *n* = 3 (55–57) demonstrated a partially mediating effect from markers of systemic inflammation (CRP, IL-6, TNF*α*) while the others (53, 54, 58) merely indicated such an effect. Two studies (53, 54) were strongly indicative, due to the controlled environment, temporal and proportional relationships between predictor, mediator, and outcome variables. There were no studies demonstrating a lack of correlation or the absence of a mediation effect. As the observed indirect effects were consistently small, the findings suggest that systemic inflammation likely operates alongside direct pathways or additional mediating variables.

In summary, the result of this systematic review suggests that systemic inflammation might be mediating a part of the relationship between altered sleep and musculoskeletal pain.

#### Obesity as the predictor variable

The most studied relationship between obesity and musculoskeletal pain proved to be osteoarthritis (OA). Among the nine studies selected for this review, five investigated OA specifically, and among these were three studies on knee OA (59, 61, 63), one on hand OA (62) and one on OA in general (67). Two of the studies on knee OA (59, 63) were of high quality and had a study design allowing conclusions to be drawn about temporality (longitudinal/intervention). Dai et al. (59) found an increased incidence of knee OA with higher body mass index (BMI) that was partially mediated by elevated CRP levels, with no association in the opposite direction (elevated CRP in response to OA). The other high-quality study on knee OA by Huebner et al. (63) was an intervention study that demonstrated reduced pain (WOMAC score), along with reduced leptin and IL-6 levels in response to weight loss. Unfortunately, this study did not measure a mediating effect of IL-6 (or leptin) on pain reduction after weight loss; however, all effects on leptin and most of the effects on IL-6 after intervention were mediated by changes in BMI.

The remaining studies on OA were cross-sectional and of moderate quality, with Yan et al. (67) demonstrating an increased risk of OA with increased BMI mediated in part by increased CRP levels. This study also demonstrated a significant serial mediation of dyslipidaemia, elevated CRP, and OA. Gløersen et al. (62) made use of several different pain measurements and markers of inflammation, where BMI was associated with all; however, only the outcome “total painful body joint count” was mediated by systemic inflammation and only in the form of high-sensitivity CRP (hsCRP). Moreover, a trend was seen for a mediating effect on “widespread pain”; however, this did not reach the level of significance. The last study on OA by Fowler-Brown et al. (61) differed from the rest in the sense that it was an elderly study population (>70 years) and the only marker of inflammation was the adipokine leptin. The results showed a correlation between BMI and knee OA and a proportional correlation with leptin levels. Leptin mediated ∼49% of the association between BMI and knee OA.

All five studies on obesity and OA are suggestive of a mediating effect from systemic inflammation, with the strongest evidence for knee OA; however only two (59, 63) provide direct evidence of causality due to their longitudinal designs.

The remaining studies on obesity had a cross-sectional design and were mainly of moderate quality. However one of these, by Perera et al. (65), was of high quality and combined a cross-sectional and longitudinal design examining the incidence of back pain. In this study, BMI and total fat mass (TFM) were associated with experiencing a back pain episode lasting more than two weeks, but no mediation effects were detected from either hsCRP or any of the inflammatory cytokines IL-6 or TNF*α*.

Two of the studies examined nonspecific or non-regional specific pain (60, 66). The study by Eslami et al. (60) found that BMI correlated with pain intensity and pain interference (SF-36 bodily pain subscale score), partly mediated by hsCRP, among elderly women. In contrast, Ray et al. (66) found that BMI and especially abdominal obesity were associated with chronic pain (TPI scores) in the elderly, but this association was not mediated by hsCRP.

Lastly, Luo et al. (64) demonstrated an association of BMI and CRP with knee pain (MPQ score). This study did not test for CRP as the mediator, but instead demonstrated that BMI mediates the association of CRP with knee pain. The opposite direction of causation is equally plausible (CRP mediating the effect of BMI on knee pain) and temporality cannot be established.

Taken together, the results regarding obesity as an influencing factor can be divided into slightly different indications for different types of pain and different populations. The strongest evidence for an association between BMI and pain is seen with OA, and especially knee OA. The findings of this review suggest that systemic inflammation mediates part, but not all, of this association. The mediating role of the adipokine leptin might be stronger than CRP in this association.

According to the single study in this review examining the association between obesity and back pain, systemic inflammation did not mediate the effect (65). For other types of pain, the findings were mixed: there were strong associations with obesity, but these were not consistently mediated by systemic inflammation, and none of them were able to measure causality due to their cross-sectional designs.

#### Psychological factors as the predictor variable

Six studies on psychological factors were included in this review. Studies in this group were generally of lower quality, and the findings were inconsistent with those on sleep and obesity. All included studies on psychological factors found associations with musculoskeletal pain, but none were able to demonstrate a mediating effect from systemic inflammation. One study indicating such a mechanism was by Andres-Rodriguez et al. (68), although it received a low quality rating. In this study, intervention with mindfulness-based stress reduction (MBSR) reduced fibromyalgia pain (FIQR score) while simultaneously preventing a drop in serum levels of the anti-inflammatory cytokine IL-10. Baseline inflammatory cytokine levels and the inflammatory/anti-inflammatory composite ratio predicted the treatment response. This suggests that MBSR might exert an effect on pain for this patient group by means of altering inflammatory pathways. Belitardo de Oliveira et al. (70) similarly found that leisure-time physical activity (LTPA) mediated the relationship between job stress (DCSQ score) and tension-type headache (TTH) through reductions in acute-phase glycoprotein (GlycA) levels. This finding indicates that physical activity has a protective effect on job stress-induced TTH by means of reducing systemic inflammation. This effect was not found for migraine, where LTPA had a protective effect independent of inflammatory markers.

However, none of these studies measured stress as a direct risk factor for pain and with a mediation analysis for markers of systemic inflammation. Two studies that were structured in this way examined depression as the predictor variable and both demonstrated similar results. In a combined cross-sectional and longitudinal study, Banafa et al. (69) found an association between depressive symptoms (BDI score) and temporomandibular (TM) pain that was not mediated by hsCRP. However, they observed an increased risk of TM pain from depressive symptoms being potentiated by elevated hsCRP. Consistent with this, Poleshuck et al. (73) demonstrated a relationship between depressive symptoms (CES-D-R score) and pain intensity and pain interference (SF-36 bodily pain subscale score) that was not mediated by serum IL-6 levels. However, they found that increased IL-6 levels correlated with more pain during depression but not in its absence. Together, these studies suggest that while systemic inflammation might not mediate a relationship between depression and pain, the influence of depression on pain seems to be aggravated by systemic inflammation.

A version of this phenomenon was also found by Graham et al. (72) in regards to chronic stress. In this study, chronic stress (being caregiver to a relative) was associated with more bodily pain (SF-36 bodily pain subscale score), not mediated by CRP; however, pain was associated with higher CRP levels for those under chronic stress, compared to controls. This result indicates either a stronger inflammatory response to pain during stress, or systemic inflammation causing more pain in the chronically stressed.

In line with these results, Dalecheck et al. (71) found that elevated CRP was predictive of chronic pain for patients with anxiety, with such an effect being significantly less pronounced for those without anxiety. This study also examined ACEs in relation to pain and systemic inflammation. Physical abuse and sexual abuse during childhood did not separately interact with CRP levels to predict chronic pain, but when both physical and sexual abuse occurred during childhood, they significantly increased the effect CRP levels had on predicting chronic pain.

Taken together, these articles do not support the notion that systemic inflammation mediates an association between psychological factors and musculoskeletal pain. However, they do suggest another interaction effect where depression, anxiety, chronic stress, and, to some extent, ACEs are associated with increased pain in the presence of systemic inflammation.

## Discussion

This systematic review lends support to small but significant mediating effects of systemic inflammation on the relationship between sleep and musculoskeletal pain. It contributes to knowledge on the relationship between obesity and musculoskeletal pain, where systemic inflammation had a small but significant mediating effect in OA, but this effect was not consistent across the few other musculoskeletal conditions studied in this review. This review does not support a mediating effect of systemic inflammation in the relationship between psychological factors and musculoskeletal pain.

Regarding sleep, we found some studies consistent with, and some in direct support for the notion that altered sleep (disruption/deprivation) had an effect on different aspects of musculoskeletal pain, partly mediated by systemic inflammation. However, mediation effects were small and several methodological limitations must be considered. First, the evidence base is characterized by heterogeneous operationalizations of both exposure and outcome measures; sleep was variously defined as reduced duration, poor subjective quality, or shift-work-related disruption, while pain outcomes ranged from site-specific intensity to generalized pressure pain thresholds. Second, the overall evidence is constrained by a scarcity of high-quality longitudinal studies. While experimental designs (53, 54, 56, 59, 63, 68, 69, 72) provide evidence for biological plausibility, the majority of the data is derived from cross-sectional designs of moderate methodological quality, which limits our ability to infer definitive mediation.

These above-mentioned limitations, combined with the small effect sizes, suggest that systemic inflammation is likely only one of several contributing mechanisms. For instance, the disturbance of endogenous pain inhibition remains a critical alternative pathway, as demonstrated by Smith et al. (74) and may operate independently of, or in parallel with, the inflammatory response.

For obesity, there was also evidence of a partially mediating effect by some inflammatory markers; however, this effect does not seem to be generalisable for different types of musculoskeletal pain. There is a strong relationship between obesity and OA, where part of the effect on weight-bearing joints is likely related to mechanical load (75). This review suggests that a part of this relationship is also mediated by systemic inflammation, although in this case, adipokines like leptin seem to be more influential than traditional markers like CRP and cytokines. In the only study investigating the association between obesity and back pain, the authors found no mediation effect of inflammatory markers (65). It is possible that both the presence and size of a mediating effect differ among inflammatory markers and that such a difference is reinforced among different painful conditions. One paradox in relation to this hypothesis is the fact that CRP is the most widely used marker of systemic inflammation, while at the same time lacking a direct mechanistic link to nociceptive stimulation (76). Inflammatory cytokines like IL-1β, TNF*α* and IL-6, together with lipid mediators such as PGE2 have several distinct ways of initiating nociceptive activity, yet many of the studies in this review measured only CRP or CRP and one specific cytokine but not others.

Three of the included studies on obesity investigated an elderly population (>70 years old) (60, 61, 66). Two of these studies demonstrated a distinct sex difference, where both BMI and markers of inflammation were related to pain (general pain/osteoarthritis) for women but not for men. It was argued that this was possibly influenced by the fact that women have more adipose tissue per unit of BMI. Older men also have higher estradiol-levels than older (post-menopausal) women, and estradiol has an inhibitory effect on inflammatory cytokines (60). One of the two studies, by Fowler-Brown et al. (61), did not investigate CRP or inflammatory cytokines, but the inflammatory adipokine leptin, which is even more closely related to adiposity. However, in this same study, there were not enough men in the sample for adequate power. It is possible that both gender and age should be considered as factors influencing the associations between obesity, systemic inflammation and pain.

The findings regarding obesity are subject to several methodological limitations. Similar to the evidence on sleep, the results are constrained by heterogeneous operationalizations of predictor, mediator and outcome variables, as well as a scarcity of high-quality longitudinal data, especially for conditions other than osteoarthritis. Another challenge in this field involves the contextualization of the inflammatory markers utilized. CRP, while frequently measured, is a non-specific marker highly sensitive to factors such as BMI, physical activity, and subclinical illness. Given the inherent association between adiposity and systemic inflammation (77), it remains difficult to definitively establish inflammation as an independent mediator.

Several studies in this review indicate that psychological factors like depression and anxiety do not *cause* pain through increased systemic inflammation. They do, however, point to an interaction effect, where psychological factors seem to have a stronger impact on pain in the presence of systemic inflammation (69, 71–73). This type of synergistic effect from psychological and immunological stressors has been found earlier in animal studies (78, 79). In humans, similar findings have been reported by Lacourt et al. (80), who demonstrated decreased pain tolerance from negative affect during systemic inflammation in healthy women. Taken together, these findings support a role for systemic inflammation in the relationship between psychological factors and musculoskeletal pain; however, not as a direct mediator of effect. Although the overall lower quality of the included studies on psychological factors warrants caution when drawing conclusions, the results across studies were consistent.

This indirect relationship between psychological factors, systemic inflammation, and musculoskeletal pain demonstrates the complexity of the interactions among all these variables and the challenges in studying them properly. Adding to the complexity, behavioural, psychological and physiological effects are probably multidirectional. For example, the relationship between depression (and anxiety) and pain is strong in both directions (i.e., pain may lead to depression and vice versa) (81). They share biological pathways and neurotransmitters, and pharmacological interventions for depression (and anxiety) can be used to treat pain (82). In the same manner, depression and anxiety are known to be associated with systemic inflammation, but systemic inflammation also increases the risk of developing depression (83, 84). It is likely that systemic inflammation, induced by any other means (such as sleep deprivation or obesity), might be a contributing factor to both depression/anxiety and pain.

While this review focused on three commonly researched factors (sleep, obesity and psychological factors), it is important to note that there are other factors, where the association with pain might be mediated by systemic inflammation. Such factors are diet (59, 85–88) and socioeconomic status/education level (44, 46), but possibly also smoking, alcohol, physical activity levels and more, as indicated by some of the studies in this review (64, 67). All these factors need further investigation in future studies before robust conclusions can be drawn.

The authors of this systematic review made an attempt to connect different influencing factors, painful conditions and measures of systemic inflammation to explore possible overarching correlations. This might not have been an optimal strategy. Though some influential factors seemingly affect pain through inflammatory activity, this probably varies with different influential factors, anatomical regions (spine, knee), tissue processes (OA, fibromyalgia) and inflammatory markers (CRP, IL-6, PGE2). Due to this variation, with only few studies available for some types of musculoskeletal pain, the interaction of systemic inflammation on a more general level is difficult to interpret. This inconsistency is also in line with the mixed findings from other authors regarding spinal pain. Klyne et al. (89) found markers of systemic inflammation to be prognostic of recovery from low back pain. Initial rises in CRP were associated with good long-term recovery, while long-term overexpression of TNF was associated with poor long-term recovery. On the other hand Suri et al. (90) failed to find a causal role for CRP in spinal pain. The extent to which separate systemic stressors can be regarded as similar in terms of effects on inflammation and pain remains unclear.

### Strengths and limitations

A strength of this study was the ability to analyse systemic inflammation as a potential mediator of the relationship between several different influential factors and musculoskeletal pain. This strength was arguably also a weakness in terms of the achievable quality of comparisons.

For example, there were considerable differences in exposure between included studies, even among those belonging to the same category (e.g., “sleep” encompassed measurements of sleep duration, forced awakenings, sleep quality scores etc). The same can be said for the outcome “musculoskeletal pain”, varying from occurrence of specific conditions such as OA, to distinct pain intensity ratings, sometimes in previously healthy (pain-free) individuals. Even the proposed mediator “systemic inflammation” was not measured by the same standards in included studies, partly owing to the fact that no such standard seems to exist. This heterogeneity between studies in terms of exposure, outcome and mediator limits the ability to draw uniform conclusions about mediating effects.

Another inherent difficulty in studying systemic inflammation as a variable, is that the most commonly measured marker, CRP, is highly sensitive to a broad spectrum of factors (BMI, infection, physical activity, smoking etc). It has been proposed to reflect any kind of (minor) metabolic stress (13, 16, 18). This is also the very reason for our interest in exploring systemic inflammation as an overarching mechanistic link between lifestyle factors and musculoskeletal pain; however, it adds to the difficulty of the task. Most of the included studies controlled for a considerable list of confounders to deal with this problem.

The use of a quality appraisal tool adapted for mediation studies (51) enabled a better assessment than the original tool developed for RCTs (50). However, the adapted version had its own limitations. As mentioned in the methods section, the assessment tool used clear-cut categories not necessarily reflecting the spectrum of possible answers in each domain. Its application was further complicated by the heterogeneity of study designs (both cross-sectional and intervention studies) and methods of measuring mediation effects. To compensate for these challenges, the authors made independent assessments of the three included articles before discussing interpretations of questions and comparing results.

To facilitate the presentation of the assessment for the reader, the quality scores were divided into groups of low, moderate and high quality, despite having no such predefined standard in the literature. As a compensatory measure, full domain-specific scores are provided in Supplementary Files S3 for transparency.

An inherent challenge in systematic reviews is the risk of selection bias. While the databases in this review were specifically chosen for their broad scope, in order not to miss relevant research, it is possible that the inclusion of other databases such as CINAHL or Web of Science, and searches targeting grey literature would have generated additional studies.

An explicit aim of this review was to examine mediation effects, rather than mere correlations between influential factors, systemic inflammation and musculoskeletal pain. Unfortunately, the incorporation of mediation effects in the research question entails challenges as to what is to be considered a mediation effect, where to draw the line and what standard to aim for in statistical analyses. In this review, any study indicating systemic inflammation as a possible link between an influencing factor and pain was first considered. The mediating effect was then evaluated through the quality assessment tool, where item number 4 directly addresses the issue of appropriate statistical methods. Methods deemed appropriate by the standard of the assessment tool were accepted at face value and, in some cases checked with a statistician. The ability to draw conclusions about *causal* mediation is specifically limited in this review, due to the inclusion of a large proportion of cross-sectional studies, lacking data on temporal relationships/directionality.

## Conclusion

The findings from this review suggest that systemic inflammation mediates part of the association between sleep and musculoskeletal pain and between obesity and OA, but not the association between psychological factors and musculoskeletal pain. Due to the heterogeneity between studies in terms of predictor, mediator and outcome variables, as well as the scarcity of high-quality longitudinal data, causal mediation cannot be confidently established. More research is required to draw firm conclusions regarding mediation, as well as to explore other influential factors and different mechanisms of associations.

## Data Availability

The original contributions presented in the study are included in the article/Supplementary Material, further inquiries can be directed to the corresponding author/s.
