# Supplementary material for: Systemic inflammation mediating the relationship between lifestyle factors and musculoskeletal pain: a systematic review
Source: Front Pain Res (Lausanne). 2026 Apr 1;7:1755744. doi: 10.3389/fpain.2026.1755744 (PMC13079645; doi:10.3389/fpain.2026.1755744)
Supplement: Supplementary file 1 [file Datasheet1.pdf]

Supplementary file 1.

Database search details

Full search in PubMed (2024-10-29)

((Sleep[Title/Abstract]) OR (Sleep[mesh]) OR (Insomnia[Title/Abstract]) OR (Insomnia[mesh]) OR (Physical activ*[Title/Abstract]) OR (Physical activ*[mesh]) OR (Physical inactiv*[Title/Abstract]) OR (Obes*[Title/Abstract]) OR (Obes*[mesh]) OR (Overweight[Title/Abstract]) OR (Overweight[mesh]) OR (Sedentary[Title/Abstract]) OR (Smok*[Title/Abstract]) OR (Smok*[mesh]) OR (Alcohol[Title/Abstract]) OR (Alcohol[mesh]) OR (Drinking[Title/Abstract]) OR (Drinking[mesh]) OR (Tobacco[Title/Abstract]) OR (Tobacco[mesh]) OR (Social isolation[Title/Abstract]) OR (Social isolation[mesh]) OR (Loneliness[Title/Abstract]) OR (Loneliness[mesh]) OR (Socioeconomic[Title/Abstract]) OR (Poverty[Title/Abstract]) OR (Poverty[mesh]) OR (Educat*[Title/Abstract]) OR (Educat*[mesh]) OR (Air pollut*[Title/Abstract]) OR (Air pollut*[mesh]) OR (Diet*[Title/Abstract]) OR (Diet*[mesh]) OR (Psych*[Title/Abstract]) OR (Psych*[mesh]) OR (Emotion*[Title/Abstract]) OR (Emotion*[mesh]) OR (Anxiety[Title/Abstract]) OR (Anxiety[mesh]) OR (Mood[Title/Abstract]) OR (Mood[mesh]) OR (Depress*[Title/Abstract]) OR (Depress*[mesh]) OR (Stress[Title/Abstract]) OR (Affective[Title/Abstract]) OR (Cognitive[Title/Abstract])) AND ((Inflammation[Title/Abstract]) OR (Inflammation[mesh]) OR (Inflammatory[Title/Abstract]) OR (Systemic inflammation[Title/Abstract]) OR (Systemic inflammation[mesh]) OR (Chronic inflammation[Title/Abstract]) OR (Chronic inflammation[mesh]) OR (Low-grade inflammation[Title/Abstract]) OR (Low-grade inflammation[mesh]) OR (Chronic low-grade inflammation[Title/Abstract]) OR (Chronic low-grade inflammation[mesh]) OR (Cytokin*[Title/Abstract]) OR (Cytokin*[mesh]) OR (Interleukin*[Title/Abstract]) OR (Interleukin*[mesh]) OR (IL-1[Title/Abstract]) OR (IL-6[Title/Abstract]) OR (IL-6[mesh]) OR (TNF[Title/Abstract]) OR (CRP[Title/Abstract]) OR (C-reactive protein[Title/Abstract]) OR (C-reactive protein[mesh])) AND ((Pain[Title/Abstract]) OR (Pain[mesh]) OR (Musculoskeletal[Title/Abstract]) OR (Somatic pain[Title/Abstract]) OR (Somatic pain[mesh]) OR (Low back pain[Title/Abstract]) OR (Low back pain[mesh]) OR (Back pain[Title/Abstract]) OR (Back Pain[mesh]) OR (Spinal pain[Title/Abstract]) OR (Spinal pain[mesh]) OR (Neck pain[Title/Abstract]) OR (neck pain[mesh]) OR (Fibromyalgia[Title/Abstract]) OR (Fibromyalgia[mesh]) OR (Osteoarthritis[Title/Abstract]) OR (Osteoarthritis[mesh]) OR (Temporomandibular[Title/Abstract]) OR (Chronic regional pain syndrome[Title/Abstract]) OR (Chronic regional pain syndrome[mesh]) OR (Myalgia[Title/Abstract]) OR (Myalgia[mesh]) OR (Tendinopathy[Title/Abstract]) OR (Tendinopathy[mesh]) OR (Myofascial[Title/Abstract]) OR (Headache[Title/Abstract]) OR (Headache[mesh]) OR (Migraine[Title/Abstract]) OR (Migraine[mesh]) OR (Subacromial[Title/Abstract]) OR (Rotator cuff[Title/Abstract]) OR (Rotator cuff[mesh]) OR (Patellofemoral[Title/Abstract]) OR (Fasciitis[Title/Abstract]) OR (Fasciitis[mesh])) AND ((Mediat*[Title/Abstract]) OR (Pathway analysis[Title/Abstract]) OR (Network analysis[Title/Abstract]) OR (Structural equation[Title/Abstract]) OR (Structural equation modeling[Title/Abstract]) OR (Structural equation modeling[mesh])) NOT ((Cancer[Title/Abstract]) OR (Dermal[Title/Abstract]) OR (Dermatitis[Title/Abstract]) OR (Dental[Title/Abstract]) OR (Dentist*[Title/Abstract]) OR (Cardiac[Title/Abstract]) OR

(Coronary[Title/Abstract]) OR (Myocardial[Title/Abstract]) OR (Heart disease[Title/Abstract]) OR (Colitis[Title/Abstract]) OR (Crohn*[Title/Abstract]) OR (Endometriosis[Title/Abstract]) OR (Rheumatoid arthritis[Title/Abstract]) OR (Psoria*[Title/Abstract]) OR (Pancreati*[Title/Abstract]) OR (Kidney[Title/Abstract]) OR (Bowel[Title/Abstract]) OR (Intestin*[Title/Abstract]) OR (Haemophilia[Title/Abstract]) OR (review[publication type]) OR (meta-analysis[Title]) OR (meta-analysis[MeSH Terms]))

Filter: Humans
Publication date: 2004-2024

Result: 1 102 articles

Full search in Scopus (2024-11-21)

(TITLE-ABS-KEY(Sleep) OR TITLE-ABS-KEY(Insomnia) OR TITLE-ABS-KEY(Physical activ*) OR TITLE-ABS-KEY(Physical inactiv*) OR TITLE-ABS-KEY(Obes*) OR TITLE-ABS-KEY(Overweight) OR TITLE-ABS-KEY(Sedentary) OR TITLE-ABS-KEY(Smok*) OR TITLE-ABS-KEY(Alcohol) OR TITLE-ABS-KEY(Drinking) OR TITLE-ABS-KEY(Tobacco) OR TITLE-ABS-KEY(Social isolation) OR TITLE-ABS-KEY(Loneliness) OR TITLE-ABS-KEY(Socioeconomic) OR TITLE-ABS-KEY(Poverty) OR TITLE-ABS-KEY(Educat*) OR TITLE-ABS-KEY(Air pollut*) OR TITLE-ABS-KEY(Diet*) OR TITLE-ABS-KEY(Psych*) OR TITLE-ABS-KEY(Emotion*) OR TITLE-ABS-KEY(Anxiety) OR TITLE-ABS-KEY(Mood) OR TITLE-ABS-KEY(Depress*) OR TITLE-ABS-KEY(Stress) OR TITLE-ABS-KEY(Affective) OR TITLE-ABS-KEY(Cognitive)) AND (TITLE-ABS-KEY(Inflammation) OR TITLE-ABS-KEY(Inflammatory) OR TITLE-ABS-KEY(Systemic inflammation) OR TITLE-ABS-KEY(Chronic inflammation) OR TITLE-ABS-KEY(Low-grade inflammation) OR TITLE-ABS-KEY(Cytokin*) OR TITLE-ABS-KEY(Interleukin*) OR TITLE-ABS-KEY(IL-1) OR TITLE-ABS-KEY(IL-6) OR TITLE-ABS-KEY(TNF) OR TITLE-ABS-KEY(CRP) OR TITLE-ABS-KEY(C-reactive protein)) AND (TITLE-ABS-KEY(Pain) OR TITLE-ABS-KEY(Musculoskeletal) OR TITLE-ABS-KEY(Somatic pain) OR TITLE-ABS-KEY(Low back pain) OR TITLE-ABS-KEY(Back pain) OR TITLE-ABS-KEY(Spinal pain) OR TITLE-ABS-KEY(Neck pain) OR TITLE-ABS-KEY(Fibromyalgia) OR TITLE-ABS-KEY(Osteoarthritis) OR TITLE-ABS-KEY(Temporomandibular) OR TITLE-ABS-KEY(Chronic regional pain syndrome) OR TITLE-ABS-KEY(Myalgia) OR TITLE-ABS-KEY(Tendinopathy) OR TITLE-ABS-KEY(Myofascial) OR TITLE-ABS-KEY(Headache) OR TITLE-ABS-KEY(Migraine) OR TITLE-ABS-KEY(Subacromial) OR TITLE-ABS-KEY(Rotator cuff) OR TITLE-ABS-KEY(Patellofemoral) OR TITLE-ABS-KEY(Fasciitis)) AND (TITLE-ABS-KEY(Mediat*) OR TITLE-ABS-KEY(Pathway analysis) OR TITLE-ABS-KEY(Network analysis) OR TITLE-ABS-KEY(Structural equation) OR TITLE-ABS-KEY(Structural equation modeling)) AND NOT (TITLE-ABS-KEY(Cancer) OR TITLE-ABS-KEY(Dermal) OR TITLE-ABS-KEY(Dermatitis) OR TITLE-ABS-KEY(Dental) OR TITLE-ABS-KEY(Dentist*) OR TITLE-ABS-KEY(Cardiac) OR TITLE-ABS-KEY(Coronary) OR TITLE-ABS-KEY(Myocardial) OR TITLE-ABS-KEY(Heart disease) OR TITLE-ABS-KEY(Colitis) OR TITLE-ABS-KEY(Crohn*) OR TITLE-ABS-KEY(Endometriosis) OR TITLE-ABS-KEY(Rheumatoid arthritis) OR TITLE-ABS-KEY(Psoria*) OR TITLE-ABS-KEY(Pancreati*) OR TITLE-ABS-KEY(Kidney) OR

TITLE-ABS-KEY(Bowel) OR TITLE-ABS-KEY(Intestin*) OR TITLE-ABS-KEY(Haemophili*) OR TITLE-ABS-KEY(rat) OR TITLE-ABS-KEY(mouse) OR TITLE-ABS-KEY(mice) OR TITLE-ABS-KEY(animal model) OR TITLE-ABS-KEY(rodent) OR TITLE-ABS-KEY(canine) OR TITLE-ABS-KEY(feline) OR TITLE-ABS-KEY(rabbit) OR TITLE-ABS-KEY(porcine) OR TITLE-ABS-KEY(bovine) OR TITLE-ABS-KEY(Review) OR TITLE-ABS-KEY(Meta-analysis) OR DOCTYPE ("Review") OR DOCTYPE ("Meta analysis"))

Filter

Document type: Article

Language: English

Year: 2004-2024

Excluded subject areas: Chemistry, Chemical engineering, Engineering, Agricultural and Biological Sciences, Computer Science, Environmental Science, Veterinary, Materials Science, Dentistry, Mathematics, Physics and astronomy, Earth and planetary sciences, Energy, Arts and humanities

Result: 1559 articles

Full search in PsycINFO (2024-12-02)

(TI("Sleep") OR AB("Sleep") OR SU("Sleep") OR TI("Insomnia") OR AB("Insomnia") OR SU("Insomnia") OR TI("Physical activity") OR AB("Physical activity") OR SU("Physical activity") OR TI("Obesity") OR AB("Obesity") OR SU("Obesity") OR TI("Overweight") OR AB("Overweight") OR SU("Overweight") OR TI("Sedentary") OR AB("Sedentary") OR SU("Sedentary") OR TI("Smoking") OR AB("Smoking") OR SU("Smoking") OR TI("Smoker") OR AB("Smoker") OR SU("Smoker") OR TI("Alcohol") OR AB("Alcohol") OR SU("Alcohol") OR TI("Drinking") OR AB("Drinking") OR SU("Drinking") OR TI("Tobacco") OR AB("Tobacco") OR SU("Tobacco") OR TI("Social isolation") OR AB("Social isolation") OR SU("Social isolation") OR TI("Loneliness") OR AB("Loneliness") OR SU("Loneliness") OR TI("Socioeconomic") OR AB("Socioeconomic") OR TI("Poverty") OR AB("Poverty") OR SU("Poverty") OR TI("Education") OR AB("Education") OR SU("Education") OR TI("Air pollution") OR AB("Air pollution") OR SU("Air pollution") OR TI("Diet") OR AB("Diet") OR SU("Diet") OR TI("Psychological") OR AB("Psychological") OR SU("Psychological") OR TI("Psychiatric") OR AB("Psychiatric") OR SU("Psychiatric") OR TI("Emotional") OR AB("Emotional") OR SU("Emotional") OR TI("Anxiety") OR AB("Anxiety") OR SU("Anxiety") OR TI("Mood") OR AB("Mood") OR SU("Mood") OR TI("Depression") OR AB("Depression") OR SU("Depression") OR TI("Depressive") OR AB("Depressive") OR SU("Depressive") OR TI("Stress") OR AB("Stress") OR SU("Stress") OR TI("Affective") OR AB("Affective") OR TI("Cognitive") OR AB("Cognitive")) AND (TI("Inflammation") OR AB("Inflammation") OR SU("Inflammation") OR

TI("Inflammatory") OR AB("Inflammatory") OR TI("Systemic inflammation") OR AB("Systemic inflammation") OR TI("Chronic inflammation") OR AB("Chronic inflammation") OR TI("Low-grade inflammation") OR AB("Low-grade inflammation") OR SU("Low-grade inflammation") OR TI("Cytokine") OR AB("Cytokine") OR TI("Interleukin") OR AB("Interleukin") OR TI("IL-1") OR AB("IL-1") OR TI("IL-6") OR AB("IL-6") OR SU("IL-6") OR TI("TNF") OR AB("TNF") OR TI("CRP") OR AB("CRP") OR TI("C-reactive protein") OR AB("C-reactive protein")) AND (TI("Pain") OR AB("Pain") OR SU("Pain") OR TI("Musculoskeletal") OR AB("Musculoskeletal") OR TI("Somatic pain") OR AB("Somatic pain") OR SU("Somatic pain") OR TI("Low back pain") OR AB("Low back pain") OR SU("Low back pain") OR TI("Back pain") OR AB("Back pain") OR SU("Back pain") OR TI("Spinal pain") OR AB("Spinal pain") OR SU("Spinal pain") OR TI("Neck pain") OR AB("Neck pain") OR SU("Neck pain") OR TI("Fibromyalgia") OR AB("Fibromyalgia") OR SU("Fibromyalgia") OR TI("Osteoarthritis") OR AB("Osteoarthritis") OR SU("Osteoarthritis") OR TI("Temporomandibular") OR AB("Temporomandibular") OR TI("Chronic regional pain syndrome") OR AB("Chronic regional pain syndrome") OR SU("Chronic regional pain syndrome") OR TI("Myalgia") OR AB("Myalgia") OR SU("Myalgia") OR TI("Tendinopathy") OR AB("Tendinopathy") OR SU("Tendinopathy") OR TI("Myofascial") OR AB("Myofascial") OR SU("Myofascial") OR TI("Headache") OR AB("Headache") OR SU("Headache") OR TI("Migraine") OR AB("Migraine") OR SU("Migraine") OR TI("Subacromial") OR AB("Subacromial") OR TI("Rotator cuff") OR AB("Rotator cuff") OR SU("Rotator cuff") OR TI("Patellofemoral") OR AB("Patellofemoral") OR TI("Fasciitis") OR AB("Fasciitis") OR SU("Fasciitis")) AND (TI("Mediate") OR AB("Mediate") OR TI("Mediation") OR AB("Mediation") OR TI("Mediates") OR AB("Mediates") OR TI("Pathway analysis") OR AB("Pathway analysis") OR TI("Network analysis") OR AB("Network analysis") OR TI("Structural equation") OR AB("Structural equation") OR TI("Structural equation modeling") OR AB("Structural equation modeling") OR SU("Structural equation modeling")) NOT (TI("Cancer") OR AB("Cancer") OR SU("Cancer") OR TI("Dermal") OR AB("Dermal") OR TI("Dermatitis") OR AB("Dermatitis") OR TI("Dental") OR AB("Dental") OR TI("Dentist") OR AB("Dentist") OR TI("Dentistry") OR AB("Dentistry") OR TI("Cardiac") OR AB("Cardiac") OR TI("Coronary") OR AB("Coronary") OR TI("Myocardial") OR AB("Myocardial") OR TI("Heart disease") OR AB("Heart disease") OR TI("Colitis") OR AB("Colitis") OR TI("Crohns disease") OR AB("Crohns disease") OR TI("Crohn’s") OR AB("Crohn’s") OR TI("Endometriosis") OR AB("Endometriosis") OR TI("Rheumatoid arthritis") OR AB("Rheumatoid arthritis") OR TI("Psoriasis") OR AB("Psoriasis") OR TI("Psoriatic") OR AB("Psoriatic") OR TI("Pancreatic") OR AB("Pancreatic") OR TI("Pancreatitis") OR AB("Pancreatitis") OR TI("Kidney") OR AB("Kidney") OR TI("Bowel") OR AB("Bowel") OR TI("Intestine") OR AB("Intestine") OR TI("Intestinal") OR AB("Intestinal") OR TI("Haemophilia") OR AB("Haemophilia") OR SU("review") OR TI("meta-analysis") OR AB("meta-analysis") OR SU("meta-analysis"))

Filter

Year: 2004-2024

Result: 83 articles
