# Supplementary material for: Systemic inflammation mediating the relationship between lifestyle factors and musculoskeletal pain: a systematic review
Source: Front Pain Res (Lausanne). 2026 Apr 1;7:1755744. doi: 10.3389/fpain.2026.1755744 (PMC13079645; doi:10.3389/fpain.2026.1755744)
Supplement: Supplementary file 2 [file Datasheet2.pdf]

Supplementary file 2.

Study quality assessment tool for observational studies

Items	Yes	No
1. Did the study cite a theoretical framework?		
2. Were the psychometric characteristics of the mediator and outcome variables reported? (Computed from the present study or a reference provided)		
3. Did the study report a power calculation? If so, was the study adequately powered to detect mediation?		
4. Were statistically appropriate/ acceptable methods of data analysis used? (This includes the product of coefficient approach with bootstrapped confidence intervals, structural equation modelling, latent growth modelling, and causal mediation analysis)		
5. Did the study ascertain whether changes in the mediating variable preceded changes in the outcome variable?		
6. Did the study ascertain whether changes in the predictor variable preceded changes in the mediator variable?		
7. Did the study control for possible confounding factors (e.g., baseline values)?		

This is an adapted version of a quality assessment tool that was designed for treatment mediation studies by Mansell et al. 2013.
