# Supplementary material for: Systemic inflammation mediating the relationship between lifestyle factors and musculoskeletal pain: a systematic review
Source: Front Pain Res (Lausanne). 2026 Apr 1;7:1755744. doi: 10.3389/fpain.2026.1755744 (PMC13079645; doi:10.3389/fpain.2026.1755744)
Supplement: Supplementary file 3 [file Supplementaryfile3.pdf]

Additional file 3.

Appraisal of methodological quality

Reference	Did the study cite a theoretical framework?	Were the psychometric characteristics of the mediator and outcome variables reported? (Computed from the present study or a reference provided)	Did the study report a power calculation? If so, was the study adequately powered to detect mediation?	Were statistically appropriate/ acceptable methods of data analysis used? (This includes the product of coefficient approach with bootstrapped confidence intervals, structural equation modelling, latent growth modelling, and causal mediation analysis)	Did the study ascertain whether changes in the mediating variable preceded changes in the outcome variable?	Did the study ascertain whether changes in the predictor variable preceded changes in the mediator variable?	Did the study control for possible confounding factors (e.g., baseline values)?
**Sleep**							
Haack et al. (53)	Yes	Yes	No	No	No	Yes	Yes
Haack et al. (54)	Yes	Yes	No	No	No	Yes	Yes
Hodges et al. (55)	Yes	Yes	No (Yes)	Yes	No	No	Yes
Irwin et al. (56)	Yes	Yes	No (Yes)	Yes	No	Yes	Yes
Matre et al. (57)	Yes	Yes	No (Yes)	Yes	No	No	No
Saravaanan et al. (58)	Yes	Yes	Yes	Yes	No	No	Yes
**Obesity**							
Dai et al. (59)	Yes	Yes	No (Yes)	Yes	Yes	Yes	Yes
Eslami et al.(60)	Yes	Yes	No (Yes)	Yes	No	No	Yes
Fowler-Brown et al. (61)	Yes	Yes	No	Yes	No	No	Yes
Gløersen et al. (62)	Yes	Yes	No (Yes)	Yes	No	No	Yes
Huebner et al. (63)	Yes	Yes	No (Yes)	Yes	No	Yes	Yes
Luo et al. (64)	Yes	Yes	No (Yes)	Yes	No	No	Yes
Perera et al. (65)	Yes	Yes	No (Yes)	Yes	Yes	No	Yes
Ray et al. (66)	Yes	Yes	No (Yes)	No	No	No	Yes
Yan et al. (67)	Yes	Yes	No (Yes)	Yes	No	No	Yes
**Psychological factors**							
Andres-Rodr. et al, (68)	Yes	Yes	No	No	No	No	Yes
Banafa et al. (69)	Yes	Yes	No (Yes)	Yes	No	No	Yes

Belitardo de O. et al. (70)	Yes	Yes	No (Yes)	Yes	No	No	Yes
Dalecheck et al. (71)	Yes	No	No (Yes)	Yes	No	No	Yes
Graham et al. (72)	Yes	Yes	No	Yes	No	No	Yes
Poleshuck et al. (73)	Yes	Yes	No	No	No	No	Yes
